# Analysis of Deposition and Diffusion of Cholesterol in Silicone Hydrogel Contact Lenses Using Confocal Microscopy

**DOI:** 10.3390/vision8030055

**Published:** 2024-09-20

**Authors:** Tomasz Suliński, Natalia Nowak, Jędrzej Szymański, Jacek Pniewski

**Affiliations:** 1 Alcon Polska, Marynarska 15, 02-674 Warsaw, Poland; 2 Faculty of Physics, University of Warsaw, Pasteura 5, 02-093 Warsaw, Poland; j.pniewski@uw.edu.pl; 3 Nencki Institute of Experimental Biology PAS, Pasteura 3, 02-093 Warsaw, Poland; n.nowak@nencki.edu.pl (N.N.);

**Keywords:** contact lenses, silicone hydrogel contact lenses, contact lens deposits, contact lens lipid deposits

## Abstract

In this study, we investigated lipid deposition and diffusion in silicone hydrogel (Si-Hy) contact lenses using confocal microscopy. Different Si-Hy lenses were analyzed to understand the interaction patterns of cholesterol with various lens materials. The results highlight significant differences in the deposition and diffusion of lipids through the lenses, revealing that some materials, such as comfilcon A, allow lipids to diffuse more freely compared to others, such as samfilcon A, which provides a greater barrier. The study also observed different morphology and movement of lipid agglomerates across the lenses and above it surfaces. These findings contribute to the understanding of lipid–lens interaction, which is important for the development of lenses with improved comfort and functionality. The research highlights the importance of considering lipid interactions in the design and selection of Si-Hy contact lenses to enhance wearer comfort and lens performance.

## 1. Introduction

Contact lenses are medical devices primarily used to correct refractive errors, with an estimated 140 million users globally as of 2013 [1]. Over decades of development, various types, materials, modifications and modalities of contact lenses have emerged. Soft lenses, particularly hydrogel (Hy) and silicone hydrogel (Si-Hy) lenses, are the most commonly fitted by Eye Care Professionals (ECPs) [2]. These lenses are classified by the ISO based on material type, water content, ionicity, or surface modification [3]. When in use, lenses are in constant contact with the eye and surrounded by the tear film, which consists of various layers including water, mucins, proteins, lipids, and electrolytes [4]. This complex environment makes lenses prone to deposition of these tear film components, referred to as endogenous deposits.

Studies show that within minutes to a few hours of exposure, contact lens materials begin to interact with the tear film, resulting in the deposition of proteins and lipids [5,6,7,8]. The nature and extent of these deposits depend on factors such as the charge, size, and hydrophobicity of the tear film components, as well as the chemical composition, water content, ionic charge, pore size, and hydrophobicity of the lens material [9,10,11,12,13,14,15,16]. Hydrogel lenses tend to attract more protein deposits, while Si-Hy lenses are more susceptible to lipid deposits. This is particularly evident with reusable lenses [17].

Understanding the interactions between contact lenses and lipids is crucial for several reasons. Lipids, including cholesterol, play a significant role in maintaining the stability and integrity of the tear film [18]. Although it might seem logical to assume that lipid buildup on contact lenses would harm wearer comfort and visual clarity, current evidence does not strongly support this assumption [17,19,20]. Research has shown only weak links between lipid deposition and wearer comfort [21], with some studies even suggesting that certain lipids might improve comfort of wearing lenses made from one material [22]. This suggests that the condition and type of tear film deposits, rather than their quantity, could be more crucial in determining lens performance. Therefore, while it is essential to study lipid–lens interactions, future research should focus on the properties of the deposited lipids and their byproducts, rather than merely their quantity, to better understand their effects on comfort and lens functionality.

Given the widespread use of Si-Hy lenses and their tendency to accumulate lipid deposits, ECPs should take these interactions into account when selecting and evaluating lenses. Survey-based research among ECPs indicates that different Si-Hy materials show varying levels of lipid deposition, which in ECPs’ opinion can affect lens performance and wearer comfort, although it is important to note that using clinical assessments it is not possible to clearly identify the type of deposit [23]. This variability underscores the importance of understanding the interactions between contact lenses and lipids, particularly cholesterol, which is one of the major components of the tear film lipid layer [24].

This study aims to analyze the deposition and diffusion of cholesterol in Si-Hy contact lenses using confocal microscopy. By examining the behavior of fluorescently labeled cholesterol on different Si-Hy lens materials, we seek to elucidate the patterns of lipid interaction with these lenses. This research contributes to a better understanding of how lipids affect Si-Hy lenses, which is crucial for developing new lens designs and care solutions.

## 2. Materials and Methods

### 2.1. Contact Lenses

Table 1 lists the contact lenses (CLs) chosen for this study. All the lenses are made from Si-Hy materials (ISO/FDA Group V) and are classified as reusable lenses, either biweekly or monthly. These lenses were selected to encompass a wide range of Si-Hy materials.

### 2.2. Sample Preparation

Each lens was taken out of the packaging solution and any remaining liquid was blotted off with a tissue. The lenses were then placed in a multi-well plate, with each well containing 2 mL of PBS–NBD–CHL with fluorescence-labeled cholesterol. To make the PBS–NBD–CHL solution, the fluorescent analogue NBD-cholesterol (22-(N-(7-nitrobenz-2-Oxa-1,3-diazol-4-yl)amino)-23,24-bisnor-5-Cholen-3β-Ol) from ThermoFisher Scientific was dissolved in dimethyl sulfoxide (DMSO), also supplied by ThermoFisher Scientific, at a concentration of 10 mg in 2 mL of DMSO, yielding a 5 mg/mL solution. This was then combined with PBS (pH 7.4) to obtain a target concentration of 0.0018 mg/mL, following Lorenz et al.’s study [25]. It is important to note that the addition of the NBD group changes the hydrophilicity [26] and size of the molecule, which could potentially alter its diffusive behavior and partitioning between the hydrophilic and hydrophobic segments of the Si-Hy polymers. While this modification may affect the results, NBD-cholesterol has been successfully used in previous studies to investigate lipid interactions with Si-Hy lens materials, providing a useful proxy for understanding cholesterol behavior in these systems [7,27]. The resulting solution was mixed thoroughly to ensure even lipid distribution. The wells were sealed with screw caps, and the lenses were left to soak in the solution for 21 ± 1 h at room temperature.

### 2.3. Microscopic Measurements

**Figure 1:**
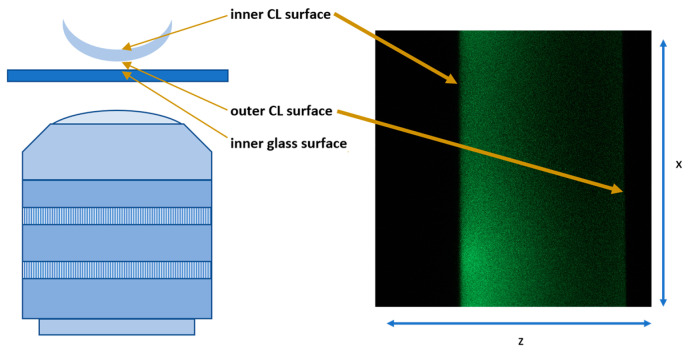
(**left**) Schematic of the microscope and samples setup, (**right**) an example of the output image.

Microscopic measurements were performed using a Leica TCS SP8 confocal microscope in an inverted configuration. The contact lenses (CLs) were placed in a 35 mm glass-bottom dish. Each lens followed the same protocol: 50 µL of PBS was first added to the bottom of the container, and the lens was placed outer surface down in the solution. Then, 100 µL of the PBS–NBD–CHL incubation solution was added to the lens. The dish containing the lens was positioned on the microscope stage. The schematic of the configuration is shown in Figure 1.

The fluorescence of NBD-cholesterol was excited by the 488 nm laser line, and its emission was detected within the 500–550 nm range using a photomultiplier tube (PMT) detector. The glass coverslip and lens surfaces were visualized with 488 nm excitation light reflected from the sample, which passed through the acousto-optical beam splitter (AOBS) set to detect reflected light. This light then reached the PMT detector in the 480–500 nm spectral range.

To locate the glass and lens surfaces, scanning was first conducted in the xz mode, where z axis was directed perpendicularly to the lens’s surface. Fluorescent lipid molecules were captured from the lens surface and matrix using the same setup (cross-sectional imaging through the lens). Once the lens’s inner surface was located, the imaging depth was adjusted accordingly, and the scanning mode switched to xyz. Images were collected within a ±10 µm range above and below the inner surface, resulting in a 20 µm thick z-stack made up of 58 xy scans of 150 µm × 150 µm with a z-step size of 0.345 µm (345 nm).

Three-dimensional models and videos were created from the collected data using Leica’s LAS X software (v. 1.4.6 28433).

To ensure that the results were not influenced by free dye, control samples for each lens material were incubated in PBS (pH 7.4) without NBD-cholesterol. These controls underwent the same fluorescence and reflection measurements as the test samples. No fluorescence signal was detected in these control lenses, and reflection measurements did not reveal any deposits on the lens surface. This confirms that the fluorescence observed in the test samples was due to the interaction of NBD-cholesterol with the lens materials and not due to free dye artifacts.

## 3. Results

**Figure 2:**
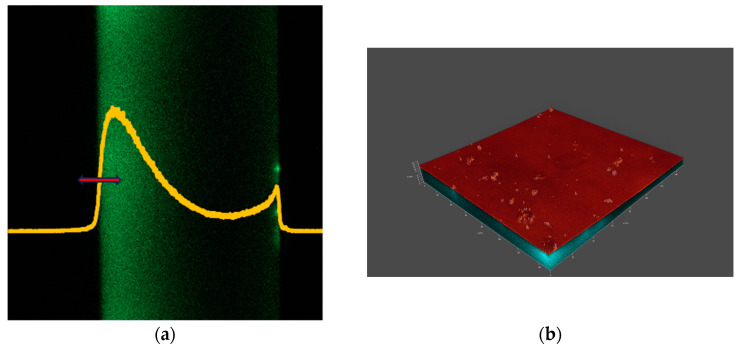
(**a**) xz and (**b**) xyz images of a CL made from senofilcon A.

**Figure 3:**
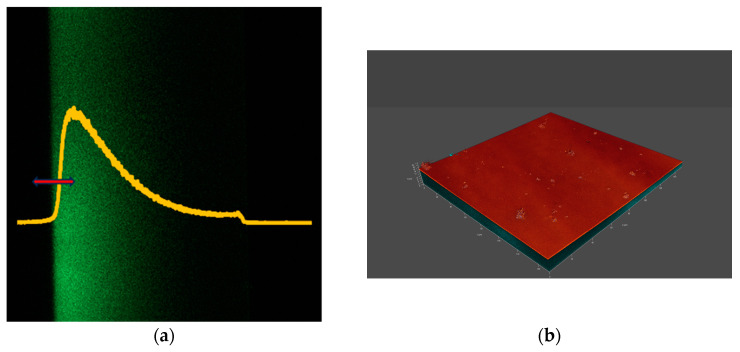
(**a**) xz and (**b**) xyz images of a CL made from lotrafilcon B.

**Figure 4:**
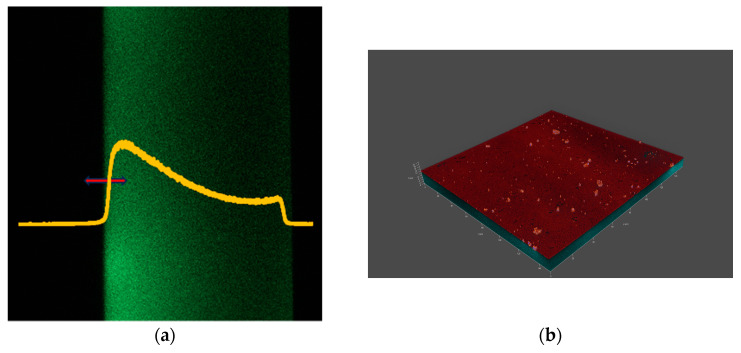
(**a**) xz and (**b**) xyz images of a CL made from comfilcon A.

**Figure 5:**
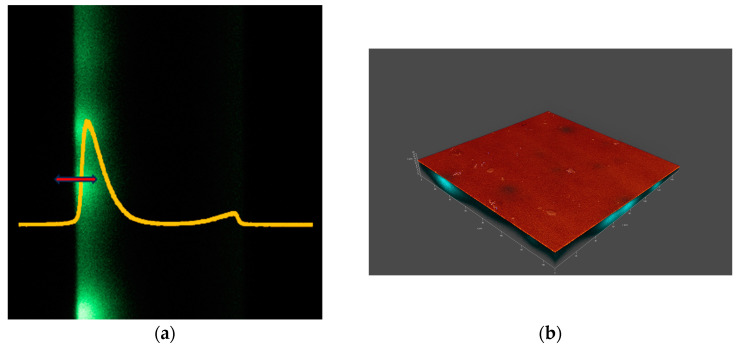
(**a**) xz and (**b**) xyz images of a CL made from Samfilcon A.

Figure 2, Figure 3, Figure 4 and Figure 5 show the microscopic measurement results. The inner surface of the lens is shown, with sections along the xz axis visible on the left panels (fluorescent signal). The intensity of the green color indicates that fluorescently labelled lipid is present on the lens surface and absorbed by the lens material. The yellow graphs show the normalized fluorescence intensity for each measurement. The red arrows indicate the measurement range along the xyz axis. The xyz measurements are shown in the accompanying figures as 3D models (a composite of xy images along the z axis), showing both fluorescence and reflectance signals. The 3D models illustrate the locations and size of agglomerates.

## 4. Limitations

While this study provides valuable insights into cholesterol deposition on Si-Hy contact lenses, several limitations should be noted.

The use of a simple NBD-cholesterol PBS solution does not fully replicate the complex tear film, which includes proteins, mucins, and electrolytes. However, more recent publications emphasize the importance of using a more complex artificial tear solution (ATS) to capture the interactions between all tear film components when demonstrating deposition profiles [7,27,28]. This simplification may lead to results that do not accurately reflect in vivo lipid deposition patterns. Future research should incorporate a more complex ATS to better mimic the tear film’s composition and interactions. 

The lenses were soaked in the cholesterol–PBS solution for 21 h under static conditions, which does not replicate the dynamic environment of the eye, including tear flow, blinking, and drying cycles. These factors could significantly impact lipid deposition and are not accounted for in this study. Future studies should consider dynamic testing methods to better simulate real-world conditions. What is more, this method does not reflect the typical daily wear (12 h) and cleaning (12 h) cycles experienced in vivo over 2–4 weeks. While the incubation period was useful for establishing baseline deposition patterns, future research should incorporate repeated wear-and-cleaning cycles to better simulate real-world conditions and provide more clinically relevant data.

Due to these limitations, the clinical applicability of the findings may be restricted. While the study offers important baseline data, additional research using more complex and dynamic models is needed to confirm these results in practical settings.

## 5. Discussion

By observing the intensity of the fluorescence signals along the z-axis, it is evident that more lipids have settled on the inner surface of the lens (visible in parts a of Figure 2, Figure 3, Figure 4 and Figure 5, as the left border of the green coloring) compared to the outer surface. However, it is important to consider that confocal microscopy has limitations, particularly in terms of reduced signal intensity when imaging through thicker media, which can also be affected by laser-induced bleaching. Additionally, while it was assumed that the lens might have adhered to the bottom of the well, it is more likely that the lens floated in the solution, which could influence the distribution and availability of lipids on the lens. For each lens, more and less intense areas can be seen, corresponding to areas of lipid agglomerate adhesion to the lens surface and lipid diffusion into the interior. This can also be seen in the 3D models (at the edges). The fluorescence intensity plots show significant differences in distribution. For example, when comparing lenses made from comfilcon A with samfilcon A, it can be seen that the former has a freer diffusion of lipids into the interior of the material. This may indicate different properties of the individual materials in terms of lipid interaction, where it can be assumed that samfilcon A provides a greater barrier to lipid diffusion than comfilcon A. The study by Rex et al. (2018) on the elemental composition of Si-Hy lens surfaces supports the idea that variations in surface chemistry, such as the presence of silicon, oxygen, and carbon, could influence how lipids like cholesterol interact with and diffuse through these materials [29]. This highlights the importance of considering both the bulk and surface properties of the lenses when analyzing lipid deposition and diffusion behavior.

Lipid adsorption on contact lens materials is influenced by several key factors, including water content, surface chemistry, and the molecular structure of the lens polymers [19]. According to the recent review by Ishihara et al. (2023), the interaction between lipids and Si-Hy lenses is largely determined by the hydrophobic and hydrophilic balance of the material, as well as the presence of surface treatments designed to reduce lipid deposition. For instance, lenses with higher water content may exhibit different lipid adsorption properties compared to those with lower water content, due to differences in hydrophilicity. Furthermore, surface modifications, such as plasma treatments, can alter the lens surface’s interaction with lipids, either enhancing or reducing adsorption [30].

**Figure 6:**
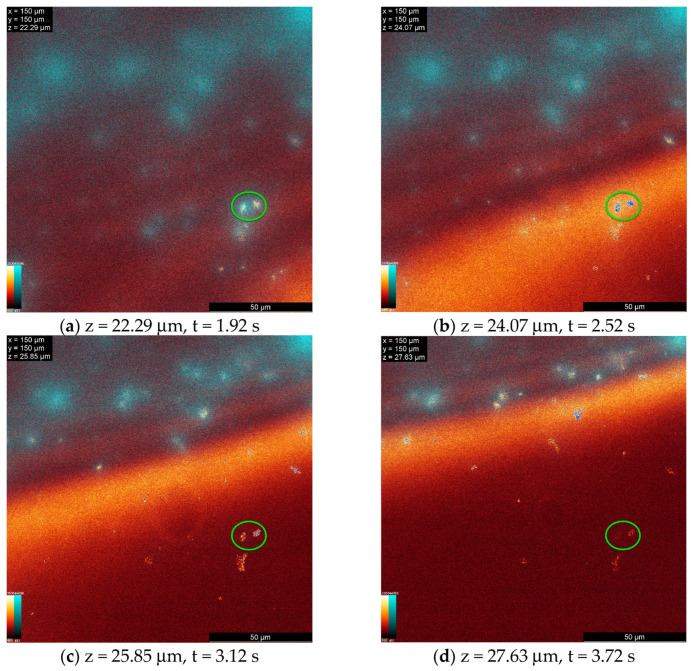
Senofilcon A. Four frames from the senofilcon A lens video representing four xy scans at different positions (z) and after different times from the start of the measurement (t).

The 3D models reveal that the morphology and number of agglomerates on the surface and within the solution differ among various lenses. For most lenses, the lipid agglomerates are relatively small and attached to the surface, as shown in Figure 6.

Videos were prepared for each lens to visualize the movement of agglomerates. Each video is a series of xy scans taken at successive z-values (from the same z-stack used for the 3D images). The first frame shows the deepest scan within the lens material, with each subsequent frame progressing along the z-axis. The frames change every 0.12 s. The videos are available as Supplementary Materials.

Figure 6 shows four frames from the video of a senofilcon A lens within a 5.34 µm space around the lens surface. The video illustrates that the lipid agglomerates permanently attach to the surface and remain immobile. The green circles highlight that agglomerates on senofilcon A lenses do not move. A correlation between the lipid deposits’ location on the surface and the fluorescence signal from the lens material is evident in the Supplementary Videos. For other lenses, some floating lipids are visible, in low number and located farther from the lens surface. A significant number of floating agglomerates can be seen above the surface of lenses made of lotrafilcon B material. This may be due to the use of plasma surface modification technology, which the manufacturer refers to as SmartShield. This technology changes the properties of the lens surface and protects it from deposits. Previous studies have shown that the lenses are particularly resistant to deposits, such as cholesterol, which is attributed to this modification [31,32,33]. Visible loose agglomerates may suggest that they will be easier for the lens wearer to remove during the lens care and cleaning procedure.

The different lipid diffusion behaviors observed in various lenses present an important but unresolved question: Is it better for a lens to exhibit high diffusivity and accumulate lipids within the lens matrix, or to have low diffusivity with lipids primarily on the surface?

On one hand, lipids accumulated on the lens surface can negatively impact wettability and potentially lead to tear film instability. Surface lipid aggregates could also increase the likelihood of deposit buildup.

On the other hand, lipids that diffuse into the lens matrix potentially could affect the bulk properties of the lens, such as oxygen permeability, light transmissibility, and water content.

At this stage, it is unclear which scenario is more detrimental to lens performance. Both surface lipid accumulation and internal lipid saturation present distinct challenges that warrant further investigation.

## 6. Conclusions

The study highlights the patterns of lipid diffusion and deposition on the surfaces and in materials of different Si-Hy lenses, indicating the variability of lipid interactions in different lens materials. 

Differences in the behavior of lipids on the lens surface and above it is demonstrated, confirming the presence and role of the modified surface of the Si-Hy lenses.

This research contributes to a better understanding of how lipid agglomerates interact with Si-Hy contact lenses, which could be important for improving lens comfort and functionality.

The differentiated absorption levels and morphological variations observed among different lens materials can inform the development of new lens designs and care solutions tailored to minimize lipid deposition.

## Figures and Tables

**Table 1:** CLs chosen for the study (H_2_O—water content, CT—central thickness, Rep—replacement modality). The power of the lenses was −3.00 dptr.

Brand Name	Material	Producer	H_2_O [%]	CT [mm]	Rep	Additional Features
Acuvue Oasys	senofilcon A	Johnson & Johnson, Jacksonville, FL, USA	38	0.07	biweekly	HYDRACLEAR^®^ PLUS Technology Class 1 UV blocker
Air Optix plus Hydraglyde	lotrafilcon B	Alcon, Fort Worth, TX, USA	33	0.08	monthly	SmartShield^®^ Technology HydraGlyde^®^ Moisture Matrix Technology
Biofinity	comfilcon A	CooperVision, Pleasanton, CA, USA	48	0.08	monthly	Aquaform^®^ Technology Aberration Neutralizing System™
Ultra	samfilcon A	Bausch & Lomb, Rochester, NY, USA	46	0.07	monthly	MoistureSeal^®^ Technology

## Data Availability

Data are available on request to the corresponding author.

## Supplementary Materials

The following supporting information can be downloaded at: https://doi.org/10.6084/m9.figshare.26131495.v2. Accessed date: 10 September 2024.
